# Lung carcinosarcoma as a rare biphasic sarcomatoid carcinoma: a case report

**DOI:** 10.4076/1757-1626-2-7968

**Published:** 2009-06-10

**Authors:** Kaushik Sanyal, Kanagasabesan Sabanathan

**Affiliations:** 1 Department of Rheumatology and General Internal Medicine, Brighton and Sussex University Hospital NHS TrustUK; 2 Department of Medicine, Norfolk and Norwich University HospitalNorwichUK

## Abstract

**Introduction:**

A fit, 57-year-old man admitted with chest pain and haemoptysis turned out to have a primary tumour in the left lung.

**Case presentation:**

In this 57-year-old Caucasian man, the diagnosis followed a computed tomography scan and histopathological evidence gained post-resection. The biopsy showed a uniform, spindle shape with focal pleomorphism which was suggestive of lung carcinosarcoma.

**Conclusions:**

The authors report this case in literature and discuss how a rare malignant tumour can be found in those presenting with trivial chest symptoms.

## Introduction

The description of lung carcinosarcoma was developed in the early 20^th^ century. It is a rare tumour, accounting for 0.3% of primary lung malignancy. The diagnosis is made through histopathological evidence. The patients presenting with this commonly undergo resection.

## Case presentation

**Figure 1.**
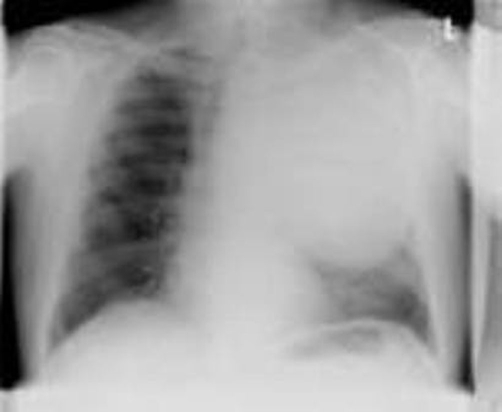
Left lung mass.

**Figure 2.**
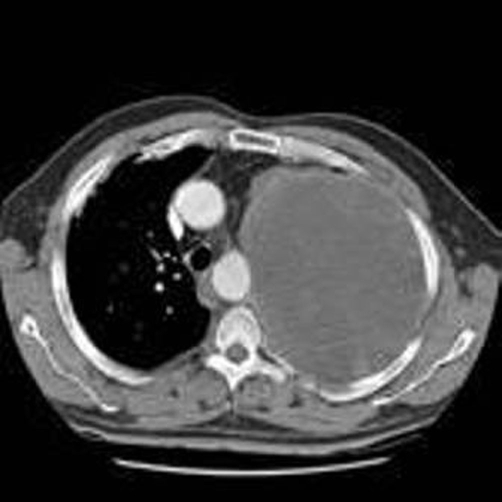
CT scan of carcinosarcoma of the left lung.

In this report, we present a rare case of a fit, 57-year-old Caucasian man who was admitted with a history of exposure to asbestosis. He presented with chest pain and frank red haemoptysis. For 2-3 weeks he had been complaining of a mild, dry cough and breathlessness on exertion. His other medical history included hypertension, gastritis and migraine. Physical examination revealed decreased air entry in the left upper lobe. The rest of the examination was normal. His chest X-ray (Figure 1) showed a large, mass-like density involving the upper-left and middle zones. Possible differential diagnoses suggested thoracic aneurysm, congenital malformation or carcinoma. A computed tomography scan showed a large, well-defined mass of mixed attenuation occupying most of the upper-left hemi-thorax, stretching mediastinal structures including the left pulmonary artery (Figure 2). There were also background changes of asbestosis. Histological examination showed mainly small, uniform spindle cells but, focally, cellular pleomorphism. There were also focal osseous and chondroid differentiations. The pattern was suggestive of sarcoma.

## Discussion and conclusion

This is a rare malignant tumour which has a mixture of carcinoma and sarcoma consisting of differentiated mesenchymal elements [1]. Regional and distant metastases are infrequent [2]. The peak age for development is in the 5^th^ to the 7^th^ decade of life [3] and there is greater preponderance in men. Final diagnosis is attainable through histological examination combined with the use of different methods, including monoclonal antibody reactions. Expression of cytokeratin and vimentin confirm the diagnosis of the tumour. This usually produces endobronchial irritation and occlusion and the location explains the symptom. Radical tumour resection and post-operative adjuvant chemotherapy are the best treatments.

## Review of current literature

Lung carcinosarcoma is a rare malignancy with a poor prognosis. It accounts for 0.3 % of pulmonary malignancies [4,5] and it is divided into endobronchial (squamous type) and peripheral (glandular type) categories. There are strong associations with smoking and asbestosis. In the endobronchial type, coughing and blood-tinged sputum usually occur; peripheral tumours are asymptomatic. WHO has added the criterion that pulmonary carcinosarcoma should show differentiation of mesenchymal components into specific tissues. Immunohistochemical differential diagnosis of sarcomatoid carcinoma of the airway in the tumour type leiomyosarcoma is: vimentin +ve, leu-7 antigen +ve, collagen type IV +ve, desmin +ve, smooth-muscle actin +ve [6].

For patients with pulmonary carcinosarcoma, the treatment of choice with a clear margin is complete resection [7]. The rate of reversibility ranged from 87-93 % [8]. Adjuvant or neoadjuvant therapy can be considered in selected cases.

## Abbreviations

None.
